# Cenário Disfuncional dos Principais Componentes Responsáveis pelo Equilíbrio do Trânsito de Cálcio Miocárdico na Insuficiência Cardíaca Induzida por Estenose Aórtica

**DOI:** 10.36660/abc.20200618

**Published:** 2021-12-20

**Authors:** Vitor Loureiro da Silva, Sérgio Luiz Borges de Souza, Gustavo Augusto Ferreira Mota, Dijon H. S. Campos, Alexandre Barroso Melo, Danielle Fernandes Vileigas, Paula Grippa Sant’Ana, Priscila Murucci Coelho, Silméia Garcia Zanati Bazan, André Soares Leopoldo, Antônio Carlos Cicogna

**Affiliations:** 1 Universidade de São Paulo Departamento de Medicina Interna Botucatu SP Brasil Universidade de São Paulo - Departamento de Medicina Interna, Botucatu, SP – Brasil; 2 Universidade Estadual Paulista São Paulo SP Brasil Universidade Estadual Paulista - Clínica Médica, São Paulo, SP – Brasil; 3 Universidade Federal do Espírito Santo Departamento de Desportos Vitória ES Brasil Universidade Federal do Espírito Santo - Departamento de Desportos, Vitória, ES – Brasil

**Keywords:** Estenose da Valva Aórtica, Insuficiência Cardíaca, Músculos Papilares, Miócitos Cardíacos, Proteínas de Ligação ao Cálcio

## Abstract

**Fundamento:**

O remodelamento cardíaco patológico se caracteriza por disfunção diastólica e sistólica, levando à insuficiência cardíaca. Neste contexto, o cenário disfuncional do trânsito de cálcio miocárdico (Ca^2+^) tem sido pouco estudado. Um modelo experimental de estenose aórtica tem sido extensamente utilizado para aprimorar os conhecimentos sobre os principais mecanismos do remodelamento patológico cardíaco.

**Objetivo:**

Entender o processo disfuncional dos principais componentes responsáveis pelo equilíbrio do cálcio miocárdico e sua influência sobre a função cardíaca na insuficiência cardíaca induzida pela estenose aórtica.

**Métodos:**

Ratos Wistar de 21 dias de idade foram distribuídos em dois grupos: controle (placebo; n=28) e estenose aórtica (EaO; n=18). A função cardíaca foi analisada com o ecocardiograma, músculo papilar isolado e cardiomiócitos isolados. No ensaio do músculo papilar, SERCA2a e a atividade do canal de Ca^2+^ do tipo L foram avaliados. O ensaio de cardiomiócitos isolados avaliou o trânsito de cálcio. A expressão proteica da proteínas do trânsito de cálcio foi analisada com o western blot. Os resultados foram estatisticamente significativos quando p <0,05.

**Resultados:**

Os músculos papilares e cardiomiócitos dos corações no grupo EaO demonstraram falhas mecânicas. Os ratos com EaO apresentaram menor tempo de pico do Ca^2+^, menor sensibilidade das miofibrilas do Ca^2+^, prejuízos nos processos de entrada e recaptura de cálcio pelo retículo sarcoplasmático, bem como disfunção no canal de cálcio do tipo L (CCTL). Além disso, os animais com EaO apresentaram maior expressão de SERCA2a, CCTL e trocador de Na^+^/Ca^2+^.

**Conclusão:**

Insuficiência cardíaca sistólica e diastólica devido à estenose aórtica supravalvular acarretou comprometimento da entrada de Ca^2+^ celular e inibição da recaptura de cálcio pelo retículo sarcoplasmático devido à disfunção no CCTL e SERCA2a, assim como mudanças no trânsito de cálcio e na expressão das principais proteínas responsáveis pela homeostase de Ca^2+^ celular.

## Introdução

A insuficiência cardíaca (IC) se caracteriza pela incapacidade do coração de realizar a perfusão de tecidos com o oxigênio e os nutrientes requeridos pela demanda metabólica do corpo;^1^ importantes desfechos intolerância ao esforço e retenção hídrica.^2^O processo da IC é produto do remodelamento mal adaptado, que pode ocorrer devido a vários tipos de dano no coração, incluindo isquemia miocárdica e sobrecarga de volume e pressão.^3^ Entre outros, o prejuízo do trânsito de cálcio é um mecanismo crucial da deterioração progressiva da função contrátil na IC.^4^

Pesquisadores reportaram mudanças na expressão e na função de proteínas reguladoras do trânsito de cálcio em várias doenças cardiovasculares.^5^ No remodelamento patológico induzido pela estenose aórtica, estudos identificaram, por meio de diferentes métodos de indução cirúrgica e períodos da doença, várias mudanças nos elementos reguladores do Ca^2^.^11,12,14^Estudos iniciais sugeriram que mudanças na saída e na entrada do retículo sarcoplasmático de Ca^2^estão relacionadas à disfunção cardíaca causada pela estenose aórtica.^11,12^ Nossos estudos anteriores avaliaram ratos com disfunção diastólica após seis e doze semanas da estenose aórtica.^16,17^ Após seis semanas, observou-se uma deficiência na atividade do cálcio ATPase (SERCA2a) do retículo endo/sarcoplasmático sem alteração na expressão da proteína;^16^após doze semanas, observou-se um aumento na fosforilação do resíduo Ser(16) da PLB e menor expressão da proteína SERCA2a.^17^ Além disso, em animais com IC após obstrução da aorta,^19,21,24^ os autores detectaram corrente de Ca^2^(I_Ca_) reduzida, ineficiência do acoplamento dos canais de cálcio do tipo L (CCTL) com os receptores Rianodina,^20^ e mudanças nas proteínas de trânsito de cálcio.^19^

Como as investigações demonstram dados diferentes relacionados ao nível estrutural e funcional do remodelamento cardíaco e as adaptações correspondentes à dinâmica do Ca^2^do miocárdio, este estudo teve como objetivo caracterizar o processo disfuncional dos principais responsáveis pelo equilíbrio do Ca^2^e sua influência na função cardíaca da IC induzida por estenose aórtica. Para este fim, diferentemente de estudos anteriores, realizamos uma avaliação cardíaca global de animais 28 semanas após a estenose aórtica. Este estudo analisou a função cardíaca nos níveis celular, tecidual e de câmara, e também examinou o trânsito de cálcio e proteínas responsáveis pelo equilíbrio do Ca^2^citosólico, apresentando resultados divergentes e surpreendentes em comparação ao que hoje está disponível na literatura.

## Métodos

### Desenho do estudo

Um grupo de ratos machos Wistar, de 21 dias de idade, foi submetido à cirurgia de indução simulada (placebo, n=22) ou de estenose aórtica (EaO, n=12). Vinte e oito semanas após o protocolo experimental, a função cardíaca foi avaliada por ecocardiograma e músculo papilar isolado. A atividade da SERCA2a e do CCTL foi analisada durante a potenciação pós-pausa e elevação do cálcio, respectivamente, e pela administração cumulativa do CA^2^ extracelular na presença de bloqueadores específicos de SERCA2a ou CCTL no ensaio do músculo papilar isolado. A expressão das proteínas reguladoras de trânsito de cálcio foi medida pelo western blot (placebo, n=7; EaO, n=7). Cinco animais com EaO foram excluídos do experimento do músculo papilar isolado por terem uma área transversal do músculo papilar maior do que 1,5 mm^2^.

Outro grupo de ratos machos Wistar foi submetido à cirurgia de indução simulada (placebo, n=6) ou de estenose aórtica (EaO, n=6). Nesses animais, o eletrocardiograma foi realizado e os cardiomiócitos foram isolados. Uma análise dos cardiomiócitos isolados foi realizada para avaliar a função mecânica dos cardiomiócitos e o manejo do cálcio.

Como observado, a avaliação do ecocardiograma foi realizada em todos os animais (placebo, n=28; EaO, n=18).

### Animais

Ratos Wistar obtidos do Centro Animal da Faculdade de Medicina de Botucatu (Botucatu, São Paulo, Brasil) foram inseridos em gaiolas coletivas a 23˚C, temperatura ambiente, em um ciclo claro-escuro de 12 horas, umidade relativa de 60% e água *ad libitum*. Esta pesquisa foi aprovada pelo Comitê de Ética em Pesquisa Experimental da Faculdade de Medicina de Botucatu – UNESP”, e pelo “Guia para o cuidado e uso de animais de laboratório” (protocolo 1138/2015).

### Cirurgia de estenose aórtica

A EaO foi induzida cirurgicamente, como já descrito.^14^ Os ratos foram anestesiados com uma mistura de cetamina (50 mg/kg, IM) e xilazina (1 mg/kg, IM), e o coração foi exposto por meio de uma toracotomia mediana. Um clip de prata (0,62 mm de diâmetro interno) foi colocado na aorta ascendente, a aproximadamente 3 mm de sua raiz, constituindo o grupo EaO (n=17). Os ratos controle foram submetidos à mesma cirurgia, porém, sem a bandagem da aorta (placebo, n=19).

### Função cardíaca

#### Ecocardiograma

Dados da estrutura cardíaca e análise de função estão expressos com variáveis do ecocardiograma 28 semanas após a estenose aórtica. Uma ecocardiografia disponível no mercado (General Electric Medical Systems, Vivid S6, Tirat Carmel, Israel), equipada com uma sonda multifrequência de 5-11,5 MHz, foi utilizada, como descrito anteriormente.^17,25,26^ Os ratos foram anestesiados por meio de uma injeção intraperitoneal, com uma mistura de cetamina (50 mg/kg) e xilazina (0,5 mg/kg). As seguintes variáveis foram utilizadas para avaliar a estrutura cardíaca: átrio esquerdo (AE) normalizado para o diâmetro da aorta (AE/Ao), diâmetro diastólico do ventrículo esquerdo (DDVE), diâmetro sistólico do ventrículo esquerdo (DSVE), espessura diastólica da parede posterior (EDPP), espessura diastólica do septo interventricular (EDSI) e espessura relativa da parede (ERP). Os seguintes parâmetros foram usados para avaliar a função ventricular: frequência cardíaca (FC), fração de encurtamento da parede média (FS); fração de ejeção (FE); velocidade sistólica da parede posterior (VSPP), velocidade de influxo mitral diastólico precoce (onda E); e pico de velocidade da onda A (contração atrial), velocidade anular mitral durante o enchimento ventricular precoce (E’), velocidade anular mitral durante a contração atrial (A’), e razão entre o fluxo de pico do enchimento e a velocidade anular mitral durante o enchimento ventricular precoce (E/E’).

#### Sinais da insuficiência cardíaca

O mesmo investigador analisou os sinais clínicos e patológicos da IC (taquipneia, ascite, efusão pleural, trombo em átrio esquerdo e hipertrofia ventricular direita), sem acesso aos grupos experimentais.

#### Ensaio isolado do músculo papilar

O desempenho contrátil do coração foi avaliado ao examinar os músculos papilares isolados do ventrículo esquerdo (VE), como descrito anteriormente.^14,17,25^ Os músculos papilares foram estimulados 12 vezes por minuto (0,2 Hz), utilizando eletrodos do tipo agulha de platina posicionados paralelamente ao eixo longitudinal dos músculos. Os eletrodos foram acoplados a um estimulador elétrico (LE12406 - Stimulator, PanLab - Harvard Apparatus, Cornella, Barcelona, Espanha) que emite estímulos de onda quadrada de 5 ms. A voltagem do estímulo utilizado foi de 12 a 15 volts, aproximadamente 10% acima do valor mínimo necessário para provocar a resposta mecânica máxima do músculo. No experimento, a solução de Krebs-Henseleit foi utilizada de acordo com a seguinte composição em mM: 118,5 NaCl; 4,69 KCl; 2,5 CaCl_2_; 1,16 MgSO_4_; 1,18 KH_2_PO_4_; 5,50 de glicose e 24,88 NaHCO_3_. A solução foi aerada por 10 minutos com oxigênio a 95% (O_2_) e dióxido de carbono a 5% (CO_2_), e mantida a 28˚C. Os seguintes parâmetros mecânicos foram medidos durante a contração isométrica: pico de tensão desenvolvida (TD; g/mm^2^), tensão em repouso (TR; g/mm2), taxa máxima da tensão desenvolvida (+tD/td; g/ mm^2^/s) e declínio (-tD/td; g/mm^2^/s), e tempo de pico de tensão (TPT; ms). Mecanismos reguladores da entrada de Ca^2^ e a atividade do CCTL foram analisados pela manobra de elevação da concentração extracelular de Ca^2^, e elevação das concentrações extracelulares de Ca^2^ (0,5, 1,5, 2,5 e 3,5 mM) na presença e na ausência de diltiazem (10^-5^M), um bloqueador específico dos CCTL. Uma manobra de potenciação pós-pausa (o estímulo foi pausado por 10, 30 e 60 s antes de reiniciar a estimulação) e uma elevação das concentrações extracelulares de Ca^2^(0,5, 1,5, 2,5, e 3,5 mM) na presença e na ausência do ácido ciclopiazônico (CPA, 30 mM), um bloqueador altamente específico de SERCA2a, foram realizadas para avaliar o potencial da função de SERCA2a. Os ensaios do bloqueio dos CCTL e SERCA2a foram avaliados com base na porcentagem de inibição, calculada como Δ(%)= (M2-M1)/M1x100, de forma que M1 é o valor da variável na concentração extracelular de cálcio, na ausência do bloqueador, e M2 é o valor da mesma variável em resposta aos bloqueadores. Testes de elevação extracelular do cálcio e potenciação pós-pausa foram analisados pelo percentual de resposta comparado à linha de base, calculada como Δ(%)= (M0-Mx)/M0×100, de forma que M0 é o valor na condição de base, e Mx é o valor absoluto em resposta à manobra (concentração aumentada de cálcio ou paralisia do estímulo elétrico). Todas as variáveis foram normalizadas por área transversal do músculo papilar. Os músculos papilares com área transversal >1,5 mm^2^ foram excluídos da análise porque podem demonstrar hipóxia central e desempenho funcional prejudicado.^16,17^

## Ensaio de cardiomiócitos isolados

### Preparação dos cardiomiócitos

Sob anestesia, ratos de cada grupo foram eutanasiados. Os corações foram rapidamente removidos por toracotomia e isolados enzimaticamente, como descrito anteriormente.^27^ Brevemente, os corações foram canulados. A perfusão retrógrada da aorta foi realizada em um sistema Lagendorff (37˚C), com solução tampão de digestão (TD) e isolamento modificada, uma solução livre de cálcio contendo 0,1 mM de etilenoglicol bis (ß-álcool aminoetílico)-N, N, N’, ácido N’-tetracético (EGTA), e N-[2-hidroxietil piperazina- N’ –[ácido 2- etanosulfónico)] (HEPES), que foram equilibradas. A composição da solução do TD foi a seguinte (mM): 130 NaCl, 1,4 MgCl_2_, 5,4 KCl, 25 HEPES, 22 de glicose, 0,33 NAH_2_PO4 e pH 7.39. Depois, os corações foram perfundidos por 15-20 minutos, com solução TD contendo 1 mg/ml de colagenase tipo II (Worthington Biochemical Corporation, Reino Unido) e Ca^2^(1 mM). Após a digestão, os corações foram removidos da cânula, cortados em pequenos pedaços e colocados em frascos cônicos com solução TD contendo colagenase suplementada com 0,1% de albumina de soro bovino e Ca^2^(1 mM). Em seguida, este processo foi realizado mais duas vezes sem a colagenase e com a adição de 1,6 e 3,12 µL de 1,0 mM da solução padrão CaCl_2_. Cada etapa contendo células e soluções foi incubada por aproximadamente 10 minutos. Então, o sobrenadante foi removido e os miócitos foram suspendidos novamente em solução de Tyrode contendo o seguinte (em mM): 140 NaCl, 10 HEPES, 0,33 NaH_2_PO_4_, 1 MgCl_2_, 5 KCl, 1,8 CaCl_2_, 10 de glicose. Somente cardiomiócitos tolerantes ao cálcio, quiescentes, em formato de haste, mostrando estriações transversais claras, foram examinados. Os cardiomiócitos isolados foram utilizados após 2 a 3 horas de isolamento.

### Contratilidade em cardiomiócitos

De forma breve, células isoladas foram colocadas em uma câmara experimental com uma base de lamelas montada na mesa de um microscópio invertido (Ion Optix, Milton, MA, EUA), com sistema de detecção de bordas e lentes objetivas 40x (Nikon Eclipse – TS100, EUA). As células foram imersas em solução de Tyrode e o campo foi estimulado a 1 Hz (20 V, 5 ms de duração dos pulsos quadrados). O encurtamento das células em resposta ao estímulo elétrico foi mensurado por meio de um sistema de detecção de bordas em vídeo, com taxa de *frames* de 240-Hz (Ionwizard, Ion Optix, Milton, MA, EUA), e os parâmetros de contração foram avaliados. O comprimento do sarcômero, o encurtamento fracional (expresso como uma porcentagem do comprimento da célula em repouso), velocidade máxima de encurtamento (VME), velocidade máxima de relaxamento (VMR), assim como tempo até o encurtamento de 50% (tempo para pico de 50%), e tempo até o relaxamento de 50% (tempo para relaxamento de 50%) foram medidos em seis células por animal em cada grupo experimental.

### Medidas de Ca2+ intracelular

Em seguida, os cardiomiócitos foram estimulados a 1Hz (Myopacer 100, Ion Optix Inc.), e as imagens fluorescentes foram obtidas com comprimento de onda de excitação alternando de 340 a 380 nm, utilizando um sistema Hyper Switch (IonOptix, Milton, MA). A emissão de fluorescência com subtração de background foi obtida, e a razão de Fura 2 AM foi utilizada como índice de transiente intracelular de [Ca^2^i, detectado em aproximadamente 510 nm. A amplitude do transiente de Ca^2^foi reportada como F/F0. F é a media de intensidade de fluorescência máxima medida no pico dos transientes de [Ca^2^i, e F0 é a intensidade base de fluorescência medida na fase diastólica dos transientes de [Ca^2^i. O tempo para o pico de [Ca^2^] e o tempo para a queda de 50% do Ca^2^também foram analisados.

## Expressão das proteínas do trânsito de cálcio

A análise western blot foi usada para avaliar a expressão da proteína dos componentes reguladores do manejo do Ca^2^. Fragmentos do VE foram congelados em nitrogênio líquido e armazenados a -80˚C. Amostras congeladas foram, então, homogeneizadas em tampão RIPA contendo inibidores de protease (Sigma-Aldrich, St. Louis, MO, EUA) e fosfatase (Roche Diagnostics, Indianápolis, IN, EUA), utilizando um homogeneizador bead beater (Bullet Blender^®^, Next Advance, Inc., NY, EUA). O produto homogeneizado foi centrifugado (5804R Eppendorf, Hamburgo, Alemanha) a 12.000 rpm por 20 minutos, a 4˚C, e o sobrenadante foi transferido para tubos Eppendorf e armazenado a -80˚C. A concentração de proteína foi determinada utilizando um kit de ensaio da proteína BCA (Pierce). A SDS-PAGE foi usada para dissolver novamente um total de 25 μg de lisado de proteína de cada amostra. A eletroforese foi realizada com gel de empilhamento bifásico (240 mm Tris-HCl pH 6,8, 30% poliacrilamida, APS e TEMED) e redissolvida (240 mm Tris-HCl pH 8,8, 30% poliacrilamida, APS e TEMED) a uma concentração de 6 a 10%, dependendo do peso molecular da proteína analisada. O Kaleidoscope Prestained Standard (Bio-Rad, Hercules, CA, EUA) foi usado para identificar o tamanho das bandas. A eletroforese foi realizada a 120 V (Power Pac HC 3.0 A, Bio-Rad, Hercules, CA, EUA) por 3 horas, com tampão (0.25 M Tris, 192 mM glicna, e 1% SDS). As proteínas foram transferidas para uma membrana de nitrocelulose (Armsham Biosciences, Piscataway, NJ, EUA), usando um sistema de mini trans-blot (Bio-Rad, Hercules, CA, EUA) com tampão de transferência (25 mM Tris, 192 mM glicina, 20% metanol e 0,1% SDS). As membranas foram bloqueadas com 5% de leite em pó desnatado em tampão TBS-T (20 mM Tris-HCl pH 7,4, 137 mM NaCl e 0,1% Tween 20) por 120 minutos, em temperatura ambiente, sob constante agitação. A membrana foi lavada três vezes com TBS-T e incubada por 12 horas a 4 – 8˚C sob constante agitação, com os seguintes anticorpos primários: *Serca2 ATPase* (1:2500; ABR, Affinity BioReagents, Golden, CO, EUA), *Fosfolambam* (1:5000; ABR), *Fosfo-Fosfolambam (Ser16)* (1:5000; Badrilla, Leeds, West Yorkshire, Reino Unido), *Fosfo–Fosfolambam (Thr17)* (1:5000; Badrilla), *Exchanger Na*^*+*^*/Ca*^2^ (1:2000; Upstate, Lake Placid, NY, EUA), *Canal de Cálcio, Voltagem Alfa 1C* (1:100; Chemicon International, Temecula, CA, EUA), *Receptor de Rianodina* (1:5000; ABR, Affinity Bioreagents, Golden, CO, EUA) e *GAPDH* (1:1000; Santa Cruz Biotechonology Inc., CA, EUA). Após a incubação com o anticorpo primário, as membranas foram lavadas três vezes em TBS-T e incubadas com anticorpos secundários conjugados com peroxidase (IgG anti-coelho ou anti-rato; 1: 5.000- 1: 10.000; Abcam) por 2 horas sob constante agitação. As membranas, então, foram lavadas três vezes com TBS-T para remover o excesso dos anticorpos secundários. Os blots foram incubados com ECL (Enhanced Chemi-Luminescence, Amersham Biosciences, Piscataway, Nova Jersey) para detecção da quimioluminescência pela ImageQuant™ LAS 4000 (GE Healthcare). A análise quantitativa dos blots foi realizada com o software Scion Image (Scion Corporation, Frederick, MD, EUA). Os imunoblots foram quantificados por densitometria utilizando o software ImageJ Analysis (NIH), e os resultados da banda alvo foram normalizados para a expressão do GAPDH do coração.^14^ Não foi possível analisar o GAPDH (37 kDa) como normalizador no mesmo gel que o receptor de rianodina (565 kDa) devido à diferença no peso molecular entre as duas proteínas. Por isso, o receptor de rianodina é expresso sem normalização.

## Análise estatística

A análise estatística foi realizada com o software Sigma Stat 3.5 (SYSTAT Software Inc., San Jose, CA, EUA). A distribuição das variáveis foi avaliada utilizando o teste de Kolmogorov-Smirnov para normalidade. De acordo com os dados de normalidade, os resultados são reportados como média ± desvio padrão (DP) ou mediana (percentil 25; percentil 75). As comparações entre os grupos foram realizadas com o teste t de Student bicaudal para amostras independentes, ou o teste te Mann-Whitney ou a análise variância com dois fatores de medidas repetidas (ANOVA), quando apropriado. O nível de significância foi considerado quando 5%.

O tamanho da amostra (n) foi estimado utilizando a equação para comparação entre grupos: n = 2SD2(Zα/2 + Zβ)2/d, no qual n é o tamanho da amostra, DP = 0,02 de estudos anteriores, Zα/2 = 1,96 (da tabela Z) com erro tipo 1 de 5%, Zβ = 0,842 (da tabela Z) com poder de 80% e = 0,02 (efeito do tamanho – diferença mínima entre os valores médios).^28^ O tamanho da amostra necessário para detectar uma diferença significativa entre os grupos foi de 16 ratos por grupo; porém, decidimos usar 22 animais com simulação (placebo) e 18 induções de estenose aórtica (EaO) por grupo para o desenho do estudo.

## Resultados

### Avaliação do ecocardiograma e sinais de insuficência cardíaca

Os dados do ecocardiograma revelaram que a estenose aórtica resultou predominantemente em hipertrofia cardíaca concêntrica (↑ERP, ↑EDPP, ↑EDSI, e ↑DDVE), dilatação do átrio esquerdo (↑AE/Ao) e disfunção diastólica (↑onda E, ↑E/A, ↑E/E’, ↓E’, e ↓A’) e sistólica (↑DSVE, ↓VEPP, ↓FS e ↓FE) 28 semanas após a cirurgia (Tabela 1). Os seguintes sinais clínicos e patológicos da IC foram detectados: ascite (30%), trombo em átrio esquerdo (48%), efusão pleural (68%), taquipneia (79%) e hipertrofia ventricular direita (100%) (Tabela 1).

**Tabela 1:** – Dados do ecocardiograma e sinais de insuficiência cardíaca

	Placebo	EaO	Valor de p
FC (bpm)	302 ± 40	298 ± 40	0,857
DDVE (mm)^*^	7,55 (7,15; 7,66)	8,43 (7,27; 9,20)	<0,001
DSVE (mm)^*^	3,20 (2,81; 3,32)	3,83 (3,32; 5,62)	<0,001
EDPP (mm)^*^	1,53 (1,53; 1,65)	2,81 (2,55; 3,07)	<0,001
EDSI (mm)^*^	1,65 (1,53; 1,70)	3,07 (2,84; 3,26)	<0,001
ERP	0,43 ± 0,03	0,69 ± 0,16	<0,001
AE/Ao	1,22 ± 0,09	1,91 ± 0,18	<0,001
Onda E (cm/s)	85 ± 7	132 ± 18	<0,001
E/A	1,49 ± 0,18	5,13 ± 1,40	<0,001
E’ (cm/s)	6,20 ± 0,78	5,32 ± 0,89	<0,001
A’ (cm/s)	4,28 ± 0,67	3,12 ± 1,18	<0,001
E/E’	13,9 ± 2,22	25,2 ± 4,66	<0,001
VSPP (cm/s)	68 ± 9	37 ± 9	<0,001
FS (%)	26,1 ± 3,42	23,3 ± 4,69	0,028
FE (%)^*^	93 (92; 94)	89 (79; 92)	<0,001
Ascite (%)	0	30	-
TAE (%)	0	48	-
EP (%)	0	68	-
Taquipneia (%)	0	79	-
HVD (%)	0	100	-

Dados são expressos em média ± DP ou mediana (percentil 25; percentil 75)^*^. Placebo: animais submetidos à cirurgia simulada (n= 28); EaO: animais submetidos à cirurgia de estenose aórtica (n= 18). FC: frequência cardíaca; DDVE: diâmetro diastólico do ventrículo esquerdo; DSVE: diâmetro sistólico do ventrículo esquerdo; EDPP: espessura diastólica da parede posterior; EDSI: espessura diastólica do septo interventricular; ERP: espessura relativa da parede no ventrículo esquerdo; AE: átrio esquerdo; AO: diâmetro da aorta; E/A: razão entre o pico de fluxo de enchimento (onda E) e pico de fluxo da contração atrial (onda A); FE: fração de ejeção; FS: fração de encurtamento da parede média; VSPP: velocidade sistólica da parede posterior; E’: velocidade anular mitral durante o enchimento ventricular precoce; A’: velocidade anular mitral durante a contração atrial; E/E’: razão entre o pico de fluxo do enchimento e a velocidade anular mitral durante o enchimento ventricular precoce. TAE: trombo em átrio esquerdo; EP: efusão pleural; HVD: hipertrofia ventricular direita. Teste t de Student ou teste de Mann-Whitney. *p < 0,05.

### Avaliação isolada do músculo papilar

#### Dados de base

A estenose aórtica prejudicou as funções de contração e relaxamento do miocárdio ao reduzir a tensão desenvolvida e a taxa máxima de tensão desenvolvida, e ao aumentar a tensão de repouso e o tempo do pico de tensão (Tabela 2).

**Tabela 2:** – Dados de base

	Placebo	EaO	Valor de p
ATV (mm^2^)	1,15 ± 0,16	1,18 ± 0,20	0,589
TD (g/mm^2^)	6,26 ± 1,58	5,18 ± 0,93	0,039
TR (g/mm^2^)	0,60 ± 0,20	0,80 ± 0,24	0,010
+tD/td (g/mm^2^/s)	66,6 ± 17,7	46,9 ± 10,3	0,001
-tD/td (g/mm^2^/s)	22,1 ± 5,24	23,9 ± 5,40	0,346
TPT (ms)^#^	180 (180; 185)	200 (180; 217)	0,007

*Os dados são expressos como média ± DP ou mediana (percentil 25; percentil 75)^#^. Placebo: animais submetidos à cirurgia simulada (n= 22); EaO: animais submetidos à cirurgia de estenose aórtica (n= 12). ATV: área transversal papilar; TD: pico de tensão desenvolvida; TR: tensão de repouso; +tD/td: taxa máxima de tensão desenvolvida; -tD/td: taxa máxima do declínio da tensão; TPT: tempo de pico de tensão. Teste t de Student ou teste de Mann-Whitney. p< 0,05.*

#### Manobras do músculo papilar isolado

**Figura 1:**
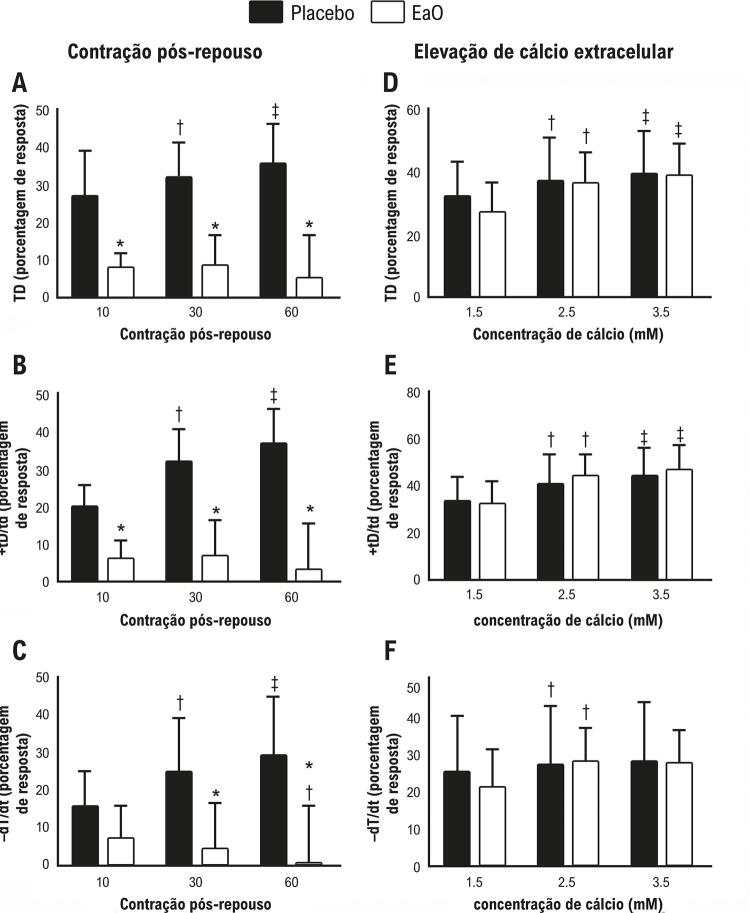
– Porcentagem de resposta à contração pós-repouso (1A, B e C) e elevação da concentração de cálcio extracelular (1D, E e F) desde a base (concentração de Ca^2+^: 0,5 mM). TD: pico de tensão desenvolvida; +tD/td: taxa máxima de tensão desenvolvida; -tD/td: taxa máxima do declínio da tensão. Os dados são expressos como media ± DP da porcentagem da manobra de resposta. Placebo: animais submetidos à cirurgia simulada (n=22). EaO: animais submetidos à cirurgia de estenose aórtica (n=12). Análise de variância para medidas repetidas e teste post-hoc de Bonferroni. *p< 0,05 vs. Placebo; †p< 0,05 vs. 10 segundos; ‡p< 0.05 vs. 10 segundos e 30 segundos (1A-C); †p< 0,05 vs. 1,5 Ca^2+^; ‡p< 0,05 vs. 1,5 e 2,5 Ca^2+^ (1D-F).

**Figura 2:**
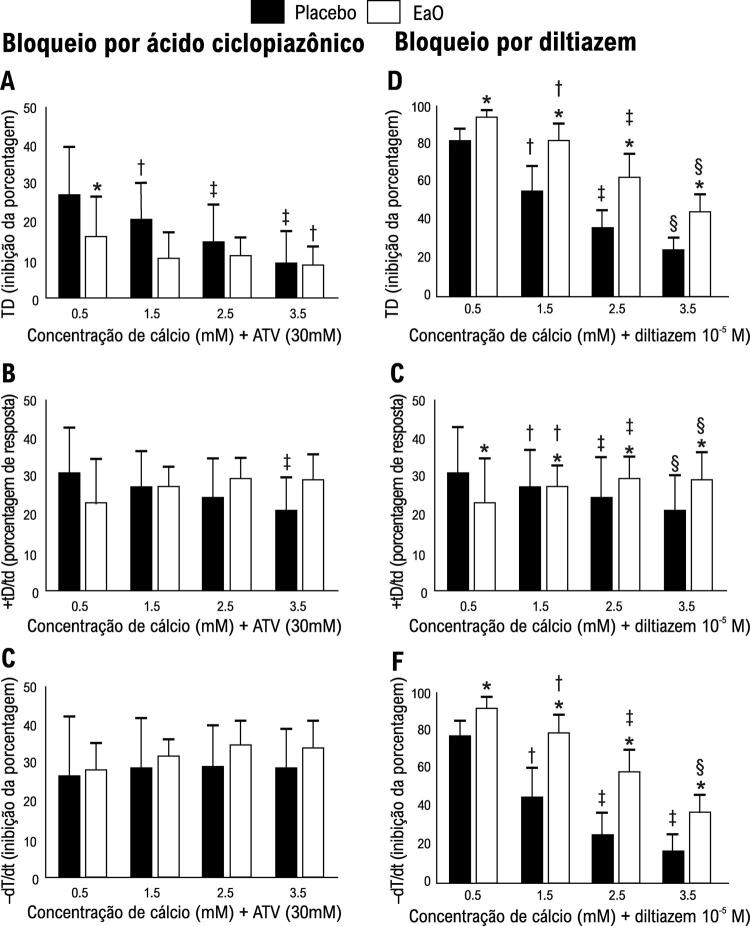
– Inibição da porcentagem de TD (pico de tensão desenvolvida), +dT/dt (taxa máxima de tensão desenvolvida) e -tD/td: taxa máxima do declínio da tensão para ácido ciclopiazônico (bloqueador de SERCA2a; figura 2A, B e C) e diltiazem (bloqueador dos canais de cálcio do tipo L); figura 2D, E e F) mais a concentração incremental de cálcio. Dados são expressos como média ± DP da porcentagem de resposta da manobra. Placebo: animais submetidos à cirurgia simulada (n=22). EaO: animais submetidos à cirurgia de estenose aórtica (n=12). Análise de variância para medidas repetidas e teste post-hoc de Bonferroni. *p< 0,05 vs. Sham; †p< 0,05 vs. 0,5 Ca^2+^; ‡p< 0,05 vs. 0,5, e 1,5 Ca^2+^; §p< 0,05 vs. 0,5, 1,5 e 2.5 Ca^2+^.

A Figura 1A-C apresenta a porcentagem de resposta do músculo papilar à potenciação pós-pausa (10, 30 e 60 s). Animais com estenose aórtica apresentaram pouca resposta à manobra de potenciação pós-pausa em comparação aos animais no placebo para todos os períodos e variáveis avaliados. Não houve diferença significativa entre os grupos em nenhum período. A Figura 1D-F apresenta a porcentagem de resposta do músculo papilar para aumentar a concentração de cálcio extracelular (1,5; 2;5 e 3,5 mM). A Figura 2A-C mostra as respostas do músculo papilar à inibição de SERCA2a e um aumento na concentração de cálcio. Após a inibição de SERCA2a pelo ácido ciclopiazônico, houve diferença significativa entre os grupos na concentração de cálcio a 0,5 mM para o pico de tensão desenvolvida (Figura 2A). A taxa máxima de tensão desenvolvida e de declínio não demonstraram diferença entre os grupos EaO e placebo. A Figura 2D-F apresenta a resposta do músculo papilar à inibição do canal de cálcio tipo L, mostrando um aumento na concentração de cálcio. Animais com estenose aórtica tiveram pior desempenho na resposta à elevação do cálcio após o bloqueio do CCTL em comparação aos animais do placebo para todos os períodos e variáveis avaliados.

## Cardiomiócitos isolados

### Função mecânica e análise do manejo do cálcio

**Figura 3:**
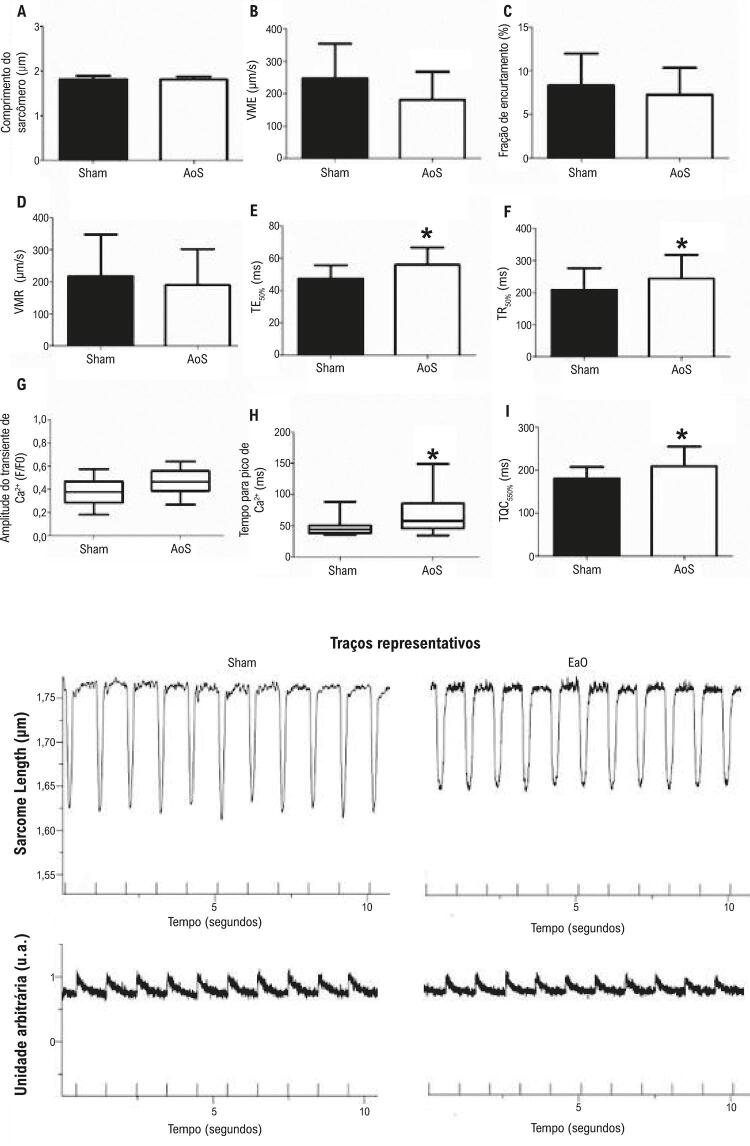
– A função mecânica dos cardiomiócitos e o manejo do cálcio do cardiomiócito. VME: velocidade máxima de encurtamento; VMR: velocidade máxima de relaxamento; TE_50%_: tempo para 50% de encurtamento; TR_50%_: tempo para 50% de relaxamento; TQC_50%_: Tempo para 50% de queda do Ca^2+^. Dados são expressos como média ± DP, ou mediana (percentil 25; percentil 75). Placebo: animais submetidos à cirurgia simulada (n=6; número de células: 36); EaO: animais submetidos à cirurgia de estenose aórtica (n=6; número de células = 36). Teste t de Student. *p< 0,05.

A Figura 3A-F mostra a função mecânica dos cardiomiócitos. A estenose aórtica afetou a VME (Figura 3B) e os tempos para alcançar 50% de contração dos cardiomiócitos (Figura 3E) e o pico de relaxamento (Figura 3F). A Figura 3G-J resume trânsito de cálcio 28 semanas após a estenose aórtica. Os animais com EaO apresentaram alterações nos tempos para alcançar o pico do Ca^2^e a queda de 50% de Ca^2^.

### Expressão das proteínas do trânsito de cálcio

**Figura 4:**
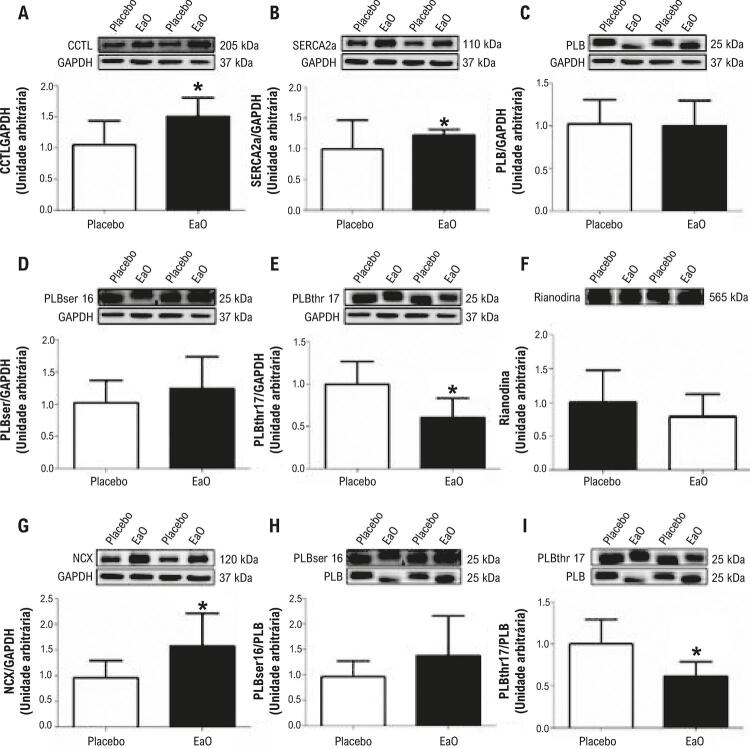
– Expressão da proteína de manejo de cálcio. Dados são expressos como média ± DP. Placebo: animais submetidos à cirurgia simulada (n=7); EaO: animais submetidos à cirurgia de estenose aórtica (n=7). CCLT: canais de cálcio do tipo L; SERCA2a: retículo sarco/endoplasmático de Ca^2+^; PLB: Fosfolamban; PLB_ser16_: fosfolambam fosforilada em serina 16; PLB_thr17_: fosfolambam fosforilada em treonina 17; NCX: antiportador Na+/Ca2+. Rianodina é expressa sem normalização. Teste t de Student. *p< 0.05.

Os dados relacionados à expressão proteica dos elementos reguladores do trânsito de cálcio são demonstrados na Figura 4A-E. A estenose aórtica aumentou o CCTL, SERCA2a e a proteína de expressão do antiportador Na^+^/Ca^2^, e reduziu a fosforilação no resíduo Thr(17) da fosfolambam fosforilada (PLB).

## Discussão

No modelo experimental da estenose aórtica supravalvular, estudos investigando o remodelamento patológico e a insuficiência cardíaca, com foco em alterações específicas do trânsito de cálcio e seus elementos reguladores, apresentaram dados escassos e uma reflexão superficial sobre os mecanismos. Assim, este trabalho realizou uma avaliação geral da função cardíaca, da dinâmica celular do Ca^2^, dos elementos reguladores do Ca^2^para elucidar o processo disfuncional dos principais componentes responsáveis pelo equilíbrio do Ca^2^e sua influência na função cardíaca na IC induzida por estenose aórtica.

Neste estudo, a estenose aórtica promoveu mudanças estruturais e disfunção ventricular, tanto diastólica quanto sistólica, como avaliado no ecocardiograma; resultados similares a estudos anteriores.^14,15,17,29^ Animais com EaO desenvolveram hipertrofia concêntrica do ventrículo esquerdo e dilatação do átrio esquerdo, características marcantes neste modelo experimental de sobrecarga de pressão cardíaca.^1,14,15,17,29^ Com base na Lei de Laplace (Estresse = Pressão x Raio/2 x Espessura), o aumento na espessura relativa da parede ventricular esquerda teve como intenção normalizar o estresse parietal sistólico devido à obstrução mecânica da aorta.^1,34,35^ Porém, a diminuição da função sistólica sugere que mesmo após o processo de hipertrofia e o estresse parietal sistólico normalizado, a redução da capacidade contrátil foi responsável pela queda no desempenho sistólico. A função mecânica dos músculos papilares e dos cardiomiócitos reproduziu respostas similares ao exame ecocardiográfico. Animais com EaO apresentaram redução e menos habilidade de desenvolver força (↓TD e +tD/td), encurtar (↓VME e ↑TE_50%_) e relaxar (↑TR e TR_50%_). Este dano funcional ao músculo papilar isolado no estágio tardio da estenose aórtica está de acordo com nosso estudo anterior, que avaliou a doença cardíaca seis semanas após cirurgia.^16^ Além disso, de acordo com os resultados dos cardiomiócitos isolados, os dados da literatura mostram que há uma redução na velocidade de encurtamento dos cardiomiócitos.^18^ Em nossos animais, a queda cardíaca funcional resultou em IC, expressa pelos seguintes sinais clínicos e patológicos: padrão de respiração alterado, ascite, efusão pleural e trombo em átrio.

Neste modelo experimental, o processo patológico do remodelamento patológico pode induzir um déficit de oxigênio como ponto de partida. A rarefação capilar do miocárdio,^22^ produto da hipertrofia da parede ventricular, pode representar a origem da patologia. Vários mecanismos podem ser estabelecidos e hiperativados para reajustar o padrão estrutural e funcional do coração, incluindo tônus simpático, o sistema renina-angiotensina-aldosterona, mediadores inflamatórios, estresse oxidativo e a regulação da expressão do gene do miocárdio via microRNAs.^36^ Porém, a ativação não harmônica desses microssistemas devido às demandas cardíacas e corporais gera diversas respostas fisiopatológicas, incluindo danos ao manejo do cálcio dos cardiomiócitos.^1,34^

A incompatibilidade do Ca^2^citosólico em cardiomiócitos é um dos mecanismos chave para o mal funcionamento do coração em resposta a vários tipos de lesão.^1,4,39^ Em modelos de IC, incluindo a estenose aórtica experimental, pesquisadores caracterizaram mudanças na expressão e função transmembrana, assim como proteínas intracelulares que regulam o trânsito de cálcio.^18,20,21,43^ Neste estudo, o aumento de SERCA2a e na expressão da proteína NCX podem trazer uma resposta adaptativa para reduzir ou evitar a sobrecarga do Ca^2^ citosólico no fim da diástole. Esta resposta foi parcialmente eficiente, já que houve prejuízo de tempo para a queda do cálcio citosólico nos cardiomiócitos isolados; além disso, mesmo com o padrão compensatório molecular, o prejuízo funcional diastólico foi verificado em um tempo de relaxamento 50% menor (TR_50%_). Como neste estudo, a literatura mostra um aumento na expressão da proteína NCX na IC.^18,44^ Porém, nossos resultados diferem com relação ao SERCA2a,^16,17^ que, em geral, não mudam ou caem neste cenário da patologia.^18,44,45^ É importante observar que a recaptação do Ca^2^pode reduzir a concentração deste íon no retículo sarcoplasmático (RS) com o passar do tempo.

Consequentemente, há uma redução na quantidade disponível para liberação,^4,44^ via Rianodina, durante a sístole no mecanismo de liberação de Ca^2^induzida pelo Ca^2^dos CCTL. Neste estudo, observamos a expressão de proteína CCTL aumentada em animais com doença cardíaca. Porém, este processo adaptativo parece ser ineficiente, já que os cardiomiócitos dos animais tornaram mais lento o tempo para atingir o pico de Ca^2^, com consequente redução da velocidade máxima de encurtamento e aumento no tempo para atingir 50% do encurtamento. De acordo com a discussão anterior, Szymanska et al.^12^ propuseram que na IC causada pela EaO há mudanças tanto na captação quanto na liberação do Ca^2^pelo RS, e que esses fatores podem contribuir para a deterioração da contração e relaxamento cardíaco.

As manobras no ensaio do músculo papilar isolado foram realizadas para verificar o dano fisiológico de dois dos principais elementos da dinâmica do Ca^2^neste processo patológico, SERCA2a e CCTL. O bloqueio do ácido ciclopiazônico e a potenciação pós-pausa foram utilizados para avaliar o potencial para recaptação do Ca^2^e a capacidade funcional de SERCA2a. Havia uma diferença na resposta entre as manobras para analisar a função de SERCA2a. A contração pós-repouso mostrou que o potencial para a recaptação do Ca^2^foi prejudicado pela estenose aórtica. Porém, após o bloqueio de SERCA2a devido ao ácido ciclopiazônico, os grupos placebo e EaO mostraram respostas similares às variáveis analisadas. Considerando que os animais com a cardiopatia demonstraram maior expressão da referida proteína, a porcentagem do bloqueio deve ter sido mais alta no grupo placebo, por ter menor quantidade de SERCA2a do que o grupo EaO. Porém, como o número de proteínas não bloqueadas no grupo EaO foi maior do que no grupo placebo, é possível considerar que este grupo remanescente de SERCA2a pós-bloqueio em animais com EaO demonstrou dano funcional.

Nossos dados estão de acordo com estudos anteriores, que sugerem a estenose aórtica como sendo indutor da deterioração funcional de SERCA2a.^11,12,16^ Além disso, nossos achados mostram que a expressão aumentada de SERCA2a, o principal mantenedor da homeostase citosólica de Ca^2^, não foi suficiente para compensar pela redução na atividade intrínseca desta proteína; esta hipótese é reforçada pelo resultados deste estudo, que demonstraram tempo reduzido para atingir 50% da queda do cálcio em cardiomiócitos. Como SERCA2a é uma ATPase, sob condições de baixa adenosina trifosfato (ATP), a atividade intrínseca desta proteína poderia ser prejudicada, causando disfunção na recaptação do Ca^2^pelo RS.^45^ Em favor desta hipótese, um estudo prévio feito pelo nosso grupo (esses resultados não estão publicados) mostrou que animais com EaO, duas semanas após cirurgia, apresentaram aumento no fator induzido por hipoxia-1 (HIF-1 α), indicador mais importante do déficit de oxigênio tecidual, que pode indicar uma redução na produção de ATP.

Além das alterações mencionadas anteriormente, este estudo identificou uma redução na forforilação de fosfolambam em treonina 17, sugerindo que o prejuízo da recaptação de Ca^2^ pode não só ser atribuído ao prejuízo funcional intrínseco de SERCA2a, mas também ao maior bloqueio desta proteína pela fosfolambam.

O bloqueio de diltiazem e a elevação de Ca^2^extracelular no músculo papilar mostrou que animais com doença cardíaca tinham a função CCTL prejudicada. Embora tenha havido um aumento na expressão de proteína desses canais, houve um aumento no tempo para alcançar o pico de Ca^2^em cardiomiócitos isolados, uma redução na VME e aumento no TE_50%_. Esses achados estão de acordo com os resultados da literatura, que mostram que o prejuízo cardíaco por EaO geram uma variante de *splicing fetal* (Ca_v_1.2_e21+22_) que reduz a expressão e a atividade desses canais, e que aumenta a ubiquitinação de CCTL via degradação proteossomal.^21^ Além disso, no remodelamento patológico pela EaO juntamente à redução de I_Ca_, há ineficiência no acoplamento de CCTLs com receptores Rianodina, tanto devido à degradação de túbulos T quanto pela redução de junctofilina-2, a proteína responsável por ancorar o retículo sarcoplasmático à membrana celular.^20^

### Limitações do estudo

Neste estudo, os cardiomiócitos não foram isolados somente no ventrículo esquerdo. Então, os resultados dos cardiomiócitos tanto dos ventrículos esquerdo como direito foram avaliados e discutidos. É importante enfatizar que um entendimento adequado da fisiologia cardíaca em estudos celulares requer conhecimento sobre a contratilidade ventricular, já que os ventrículos direito e esquerdo têm propriedades funcionais distintas. Assim, também seria relevante realizar uma análise molecular em ambos os ventrículos; porém, a expressão das proteínas do trânsito de cálcio apenas foi realizada no VE.

## Conclusões

Nosso estudo buscou esclarecer e facilitar o entendimento sobre os eventos fisiopatológicos do trânsito de cálcio e sobre as mudanças em seus agentes reguladores principais no processo patológico cardíaco causado pela estenose aórtica. De acordo com nossos resultados, neste modelo experimental de IC, há mudanças relevantes na dinâmica do cálcio devido a alterações na expressão de NCX e SERCA2a, na expressão da proteína do CCTL, e redução da fosforilação do resíduo Thr(17) de PLB. Além disso, o dano funcional da SERCA2a e do CCTL foram essenciais para a deterioração contrátil e do relaxamento. Assim, é importante desenvolver tratamentos que foquem não só em SERCA2a e CCTL, mas também no entendimento de todos os processos patológicos para reequilibrar o fluxo do cálcio intracelular e a função cardíaca.
