# Supplementary material for: Cenário Disfuncional dos Principais Componentes Responsáveis pelo Equilíbrio do Trânsito de Cálcio Miocárdico na Insuficiência Cardíaca Induzida por Estenose Aórtica
Source: Arq Bras Cardiol. 2021 Dec 20;118(2):463–75. [Article in Portuguese] doi: 10.36660/abc.20200618 (PMC8856692; doi:10.36660/abc.20200618)
Supplement: Translation

# Translation: The Dysfunctional Scenario of the Major Components Responsible for Myocardial Calcium Balance in Heart Failure Induced by Aortic Stenosis

**Authors:** Vitor Loureiro da Silva, Sérgio Luiz Borges de Souza, Gustavo Augusto Ferreira Mota, Dijon H. S. Campos, Alexandre Barroso Melo, Danielle Fernandes Vileigas, Paula Grippa Sant’Ana, Priscila Murucci Coelho, Silméia Garcia Zanati Bazan, André Soares Leopoldo, Antônio Carlos Cicogna

**Affiliations:** 1 Brazil Universidade de São Paulo - Departamento de Medicina Interna, Botucatu, SP – Brazil; 2 Brazil Universidade Estadual Paulista - Clínica Médica, São Paulo, SP – Brazil; 3 Brazil Universidade Federal do Espírito Santo - Departamento de Desportos, Vitória, ES – Brazil

**Keywords:** aortic stenosis, heart failure, papillary muscle, isolated cardiomyocytes, calcium handling proteins

## Abstract

**Background:**

Maladaptive cardiac remodelling is characterized by diastolic and systolic dysfunction, culminating in heart failure. In this context, the dysfunctional scenario of cardiac calcium (Ca^2+^) handling has been poorly studied. An experimental model of aortic stenosis has been extensively used to improve knowledge about the key mechanisms of cardiac pathologic remodelling.

**Objective:**

To understand the dysfunctional process of the major components responsible for Ca^2+^ balance and its influence on cardiac function in heart failure induced by aortic stenosis.

**Methods:**

Male 21-day-old Wistar rats were distributed into two groups: control (sham; n= 28) and aortic stenosis (AoS; n= 18). Cardiac function was analysed by echocardiogram, isolated papillary muscle, and isolated cardiomyocytes. In the papillary muscle assay, SERCA2a and L-type Ca^2+^ channel activity was evaluated. The isolated cardiomyocyte assay evaluated Ca^2+^ handling. Ca^2+^ handling protein expression was analysed by western blot. Statistical significance was set at p <0.05.

**Results:**

Papillary muscles and cardiomyocytes from AoS hearts displayed mechanical malfunction. AoS rats presented a slower time to the Ca^2+^ peak, reduced Ca^2+^ myofilament sensitivity, impaired sarcoplasmic reticulum Ca^2+^ influx and reuptake ability, and SERCA2a and L-type calcium channel (LTCC) dysfunction. Moreover, AoS animals presented increased expression of SERCA2a, LTCCs, and the Na^+^/Ca^2+^ exchanger.

**Conclusion:**

Systolic and diastolic heart failure due to supravalvular aortic stenosis was paralleled by impairment of cellular Ca^2+^ influx and inhibition of sarcoplasmic reticulum Ca^2+^ reuptake due to LTCC and SERCA2a dysfunction, as well as changes in Ca^2+^ handling and expression of the major proteins responsible for cellular Ca^2+^ homeostasis.

## Introduction

Heart failure (HF) is characterized by the heart’s inability to perfuse tissues with oxygen and nutrients required by the body’s metabolic demand;^1^ major outcomes include exercise intolerance and water retention.^2^The process of HF is the product of *maladaptive* remodelling, which may occur due to several types of damage to the heart, including myocardial ischaemia and volume and pressure overload.^3^ Among others, impaired calcium (Ca^2^) handling is a crucial mechanism of the progressive deterioration of contractile function in HF.^4^

Researchers have reported changes in the expression and function of regulatory proteins of Ca^2^ handling in a variety of cardiovascular diseases.^5^ In pathological remodelling induced by aortic stenosis, studies have identified, using different methods of surgical induction and periods of the disease, several distinct changes in the regulatory elements of Ca^2^.^11,12,14^ Initial studies have proposed that changes in sarcoplasmic reticulum Ca^2^ outflow and intake are related to cardiac dysfunction due to aortic stenosis.^11,12^ Our previous studies evaluated rats with diastolic dysfunction after six and twelve weeks of aortic stenosis.^16,17^ After six weeks, impairment in sarco/endoplasmic reticulum calcium ATPase (SERCA2a) activity without protein expression alteration was observed;^16^after twelve weeks, increased phosphorylation of the Ser(16) residue of PLB and reduced SERCA2a protein expression were observed.^17^ Furthermore, in animals with HF after aortic obstruction,^19,21,24^ the authors detected reduced Ca^2^ current (I_Ca_),^21^inefficiency of the coupling of the LTCCs with the RyR receptors,^20^ and alterations in calcium handling proteins.^19^

Because these investigations report scattered data regarding the structural and functional degree of cardiac remodelling and the corresponding adaptations of myocardial Ca^2^ dynamics, this study aimed to characterize the dysfunctional process of the major components responsible for Ca^2^ balance and its influence on cardiac function in HF induced by aortic stenosis. For this purpose, unlike previous studies, we performed a global cardiac evaluation of the animals after 28 weeks of aortic stenosis. This study performed cardiac function analysis at the cellular, tissue, and chamber levels, as well as examining Ca^2^ handling and the proteins responsible for cytosolic Ca^2^ balance, presenting divergent and surprising results compared to the currently available literature.

## Methods

### Study design

1) A group of twenty one-day-old male Wistar rats was submitted to simulated (Sham, n= 22) or aortic stenosis induction (AoS, n= 12) surgery. After 28 weeks of the experimental protocol, cardiac function was evaluated by echocardiogram and isolated papillary muscle. SERCA2a and L-type calcium channel activity was analysed during postrest contraction and calcium elevation, respectively, and by the cumulative administration of extracellular Ca^2^ in the presence of SERCA2a- or L-type calcium channel-specific blockers in the isolated papillary muscle assay. Expression of Ca^2^-handling regulatory proteins was measured by western blotting (sham, n= 7; AoS, n= 7). Five AoS animals were excluded from the isolated papillary muscle experiment due to the having a cross-sectional area of papillary muscle greater than 1.5 mm^2^.

2) Another group of twenty one-day-old male Wistar rats underwent simulated (Sham, n= 6) or aortic stenosis induction (AoS, n= 6) surgery. In these animals, electrocardiogram and was performed and cardiomyocytes were isolated. Analysis of the isolated cardiomyocytes was performed to assess the mechanical function of the cardiomyocytes and calcium handling.

As noted, echocardiogram evaluation was performed on all study animals (Sham, n= 28; AoS, n= 18).

### Animals

Wistar rats obtained from the Animal Center of Botucatu Medical School (Botucatu, São Paulo, Brazil) were allocated to collective cages in a 23°C room temperature with a 12-h light/dark cycle, a relative humidity of 60%, and water ad libitum. This research was approved by the “Committee for Experimental Research Ethics of the Faculty of Medicine in Botucatu – UNESP” and by the “Guide for the Care and Use of Laboratory Animals” (protocol 1138/2015).

### Aortic stenosis surgery

AoS was surgically induced as previously described.^14^ Rats were anaesthetized using a mixture of ketamine (50 mg/kg, IM) and xylazine (1 mg/kg, IM), and the heart was exposed via a median thoracotomy. A silver clip (0.62 mm internal diameter) was then placed on the ascending aorta approximately 3 mm from its root, constituting the AoS group (n= 17). Control rats were submitted to the same surgery but without aortic banding (Sham, n= 19).

### Cardiac function

#### Echocardiogram

Data from the cardiac structure and function analysis are expressed utilizing echocardiogram variables after 28 weeks of aortic stenosis. Commercially available echocardiography (General Electric Medical Systems, Vivid S6, Tirat Carmel, Israel) equipped with a 5-11.5 MHz multifrequency probe was used as previously described.^17,25,26^ Rats were anaesthetized via intraperitoneal injection with a mixture of ketamine (50 mg/kg) and xylazine (0.5 mg/kg). The following variables were used to evaluate cardiac structure: LA normalized to the aortic diameter (LA/Ao), left ventricle diastolic diameter (LVDD), left ventricular systolic diameter (LVSD), posterior wall diastolic thickness (PWDT), interventricular septum diastolic thickness (ISDT), and relative wall thickness (RWT). The following parameters were used to assess ventricular function: heart rate (HR), mid-wall fraction shortening (FS); ejection fraction (EF); posterior wall systolic velocity (PWSV), early diastolic mitral inflow velocity (E wave), the ratio between E wave and atrial contraction flow peak (A wave), the velocity of the mitral annulus during early ventricular filling (E’), mitral velocity annulus during atrial contraction (A’), and the ratio between filling flow peak and mitral annulus velocity during early ventricular filling (E/E’).

#### Heart failure signs

The same investigator analysed clinical and pathological signs of HF (tachypnoea, ascites, pleural effusion, left atrium thrombi, and right ventricular hypertrophy) and was blinded to the experimental groups.

#### Isolated papillary muscle assay

Cardiac contractile performance was evaluated by examining isolated papillary muscles from the LV, as previously described.^14,17,25^ The papillary muscles were stimulated 12 times per minute (0.2 Hz) using platinum needle-type electrodes positioned parallel to the muscles’ longitudinal axis. The electrodes were coupled to an electrical stimulator (LE12406 - Stimulator, PanLab - Harvard Apparatus, Cornella, Barcelona, Spain) that emits 5 ms square wave stimuli. The stimulus voltage used was 12 to 15 volts, approximately 10% above the minimum value necessary to provoke the muscle’s maximum mechanical response. In the experiment, Krebs-Henseleit solution was used according to the following composition in mM: 118.5 NaCl; 4.69 KCl; 2.5 CaCl_2_; 1.16 MgSO_4_; 1.18 KH_2_PO_4_; 5.50 glucose and 24.88 NaHCO_3_. The solution was aerated for 10 minutes with 95% oxygen (O_2_) and 5% carbon dioxide (CO_2_) and kept at 28°C. The following mechanical parameters were measured during isometric contraction: maximum developed tension (DT; g/mm^2^), resting tension (RT; g/mm^2^), maximum rate of tension development (+dT/dt; g/mm^2^/s) and decline (-dT/dt; g/mm^2^/s), and time-to-peak tension (TPT; ms). Regulatory mechanisms of Ca^2^ influx and L-type calcium channel activity were analysed by the Ca^2^ concentration extracellular elevation manoeuvre and elevation of extracellular Ca^2^ concentrations (0.5, 1.5, 2.5, and 3.5 mM) in the presence and absence of diltiazem (10^-5^M), a specific blocker of L-type calcium channels. A postrest contraction manoeuvre (the stimulus was paused for 10, 30, and 60 s before restarting the stimulation) and elevation of extracellular Ca^2^ concentrations (0.5, 1.5, 2.5, and 3.5 mM) in the presence and absence of cyclopiazonic acid (CPA, 30 mM), a highly specific blocker of SERCA2a, were performed to assess the potential of SERCA2a function. L-channel and SERCA2a calcium channel blocking assays were analysed from the percentage of inhibition, calculated as Δ(%)= (M2-M1)/M1x100, so that M1 is the value of the variable in the extracellular calcium concentration in the absence of the blocker, and M2 is the value of the same variable in response to the blockers. Tests of extracellular calcium elevation and postrest contraction were analysed by the percent response compared to baseline, calculated as Δ(%)= (M0-Mx)/M0×100, so that M0 is the value in the baseline condition, and Mx is the absolute value in response to the manoeuvre (increased calcium concentration or paralysis of the electrical stimulus). All variables were normalized per cross-sectional area (CSA) of the papillary muscle. Papillary muscles with CSA >1.5 mm^2^ were excluded from analysis because they can exhibit central core hypoxia and impaired functional performance.^16,17^

## Isolated cardiomyocyte assay

### Cardiomyocyte preparation

Under anaesthesia, rats from each group were euthanized. Hearts were quickly removed by median thoracotomy and enzymatically isolated, as previously described.^27^ Briefly, the hearts were cannulated. Retrograde perfusion of the aorta was performed on a Langendorff system (37°C) with a modified isolation digestion buffer solution (DB), a calcium-free solution containing 0.1 mM ethylene glycol-bis (ß-aminoethyl ether)-N, N, N’, N’-tetraacetic acid (EGTA), and N-[2-hydro-ethyl]-piperazine-N’-[2-ethanesulfonic acid] (HEPES), which were equilibrated. The composition of DB solution was as follows (mM): 130 NaCl, 1.4 MgCl_2_, 5.4 KCl, 25 HEPES, 22 glucose, 0.33 NAH_2_PO4, and pH 7.39. Afterward, the hearts were perfused for 15-20 minutes with a DB solution containing 1 mg/ml collagenase type II (Worthington Biochemical Corporation, UK) and Ca^2^(1 mM). Digested hearts were then removed from the cannula, minced into small pieces, and placed into small conical flasks with DB solution containing collagenase supplemented with 0.1% bovine serum albumin and Ca^2^(1 mM). Next, this process was performed two more times without collagenase and with the addition of 1.6 and 3.12 µL of 1.0 mM CaCl_2_ stock solution. Each stage containing cells and solutions was incubated for approximately 10 minutes. Then, the supernatant was removed, and the myocytes were resuspended in Tyrode’s buffer containing the following (in mM): 140 NaCl, 10 HEPES, 0.33 NaH_2_PO_4_, 1 MgCl_2_, 5 KCl, 1.8 CaCl_2_, 10 glucose. Only calcium-tolerant, quiescent, rod-shaped cardiomyocytes showing clear cross-striations were examined. The isolated cardiomyocytes were used within 2-3 h of isolation.

### Cardiomyocyte contractility

Briefly, isolated cells were placed in an experimental chamber with a glass coverslip base mounted on the stage of an inverted microscope (Ion Optix, Milton, MA, USA) edge detection system with a 40x objective lens (Nikon Eclipse – TS100, USA). Cells were immersed in Tyrode’s solution and field stimulated at 1 Hz (20 V, 5 ms duration square pulses). Cell shortening in response to electrical stimulation was measured using a video-edge detection system at a 240-Hz frame rate (Ionwizard, Ion Optix, Milton, MA, USA), and the contractile parameters were evaluated. Sarcomere length, fractional shortening (expressed as a percentage of resting cell length), maximum shortening velocity, maximum relaxation velocity, and time to 50% shortening (time to 50% peak) and 50% relaxation (time for 50% relaxation) were measured in 6 cells per animal in each experimental group.

### Intracellular Ca2+ measurements

Subsequently, cardiomyocytes were stimulated at 1 Hz (Myopacer 100, Ion Optix Inc.), and fluorescence images were obtained with alternating excitation at 340 to 380 nm wavelengths using a Hyper Switch system (IonOptix, Milton, MA). Background-subtracted fluorescence emission was obtained, and the Fura 2 AM ratios were used as an index of intracellular [Ca^2^i transient, which was detected at approximately 510 nm. The Ca^2^transient amplitude was reported as F/F0. F is the maximal fluorescence intensity average measured at the peak of [Ca^2^i transients, and F0 is the baseline fluorescence intensity measured at the diastolic phase of [Ca^2^i transients. The time to Ca^2^peak and time to 50% Ca^2^ decay were also analysed.

## Expression of calcium handling proteins

Western blot analysis was used to evaluate protein expression of the regulatory components of Ca^2^ handling. Fragments of the LV were frozen in liquid nitrogen and stored at -80°C. Frozen samples were subsequently homogenized in RIPA buffer containing protease (Sigma-Aldrich, St. Louis, MO, USA) and phosphatase (Roche Diagnostics, Indianapolis, IN, USA) inhibitors using a bead beater homogenizer (Bullet Blender^®^, Next Advance, Inc., NY, USA). The homogenized product was centrifuged (5804R Eppendorf, Hamburg, Germany) at 12,000 rpm for 20 minutes at 4°C, and the supernatant was transferred to Eppendorf tubes and stored at -80°C. Protein concentration was determined using the Pierce BCA Protein Assay Kit. SDS-PAGE was used to resolve a total of 25 μg protein lysate from each sample. Electrophoresis was performed with biphasic gel stacking (240 mm Tris-HCl pH 6.8, 30% polyacrylamide, APS and TEMED) and resolving (240 mm Tris-HCl pH 8.8, 30% polyacrylamide, APS and TEMED) at a concentration of 6 to 10%, depending on the molecular weight of the analysed protein. The Kaleidoscope Prestained Standard (Bio-Rad, Hercules, CA, USA) was used to identify band sizes. Electrophoresis was performed at 120 V (Power Pac HC 3.0 A, Bio-Rad, Hercules, CA, USA) for 3 h with running buffer (0.25 M Tris, 192 mM glycine, and 1% SDS). Proteins were transferred to a nitrocellulose membrane (Armsham Biosciences, Piscataway, NJ, USA) using a Mini Trans-Blot (Bio-Rad, Hercules, CA, USA) system with transfer buffer (25 mM Tris, 192 mM glycine, 20% methanol, and 0,1% SDS). Membranes were blocked with 5% non-fat dry milk in TBS-T buffer (20 mM Tris-HCl pH 7.4, 137 mM NaCl, and 0.1% Tween 20) for 120 minutes at room temperature under constant agitation. The membrane was washed three times with TBS-T and incubated for 12 h at 4 - 8°C under constant agitation with the following primary antibodies: *Serca2 ATPase* (1:2500; ABR, Affinity BioReagents, Golden, CO, USA), *Phospholamban* (1:5000; ABR), *Phospho-Phospholamban (Ser16)* (1:5000; Badrilla, Leeds, West Yorkshire, UK), *Phospho–Phospholamban (Thr17)* (1:5000; Badrilla), *Exchanger Na*^*+*^*/Ca*^2^ (1:2000; Upstate, Lake Placid, NY, USA), *Calcium Channel, Voltage-Gated Alpha 1C* (1:100; Chemicon International, Temecula, CA, USA), *Ryanodine Receptor* (1:5000; ABR, Affinity Bioreagents, Golden, CO, USA), and *GAPDH* (1:1000; Santa Cruz Biotechonology Inc., CA, USA). After incubation with the primary antibody, membranes were washed three times in TBS-T and incubated with peroxidase-conjugated secondary antibodies (anti-rabbit or anti-mouse IgG; 1: 5,000- 1: 10,000; Abcam) for 2 h under constant agitation. Membranes were then washed three times with TBS-T to remove excess secondary antibody. Blots were incubated with ECL (Enhanced Chemi-Luminescence, Amersham Biosciences, Piscataway, NJ) for chemiluminescence detection by ImageQuant™ LAS 4000 (GE Healthcare). Quantification analysis of blots was performed using Scion Image software (Scion Corporation, Frederick, MD, USA). The immunoblots were quantified by densitometry using ImageJ Analysis software (NIH), and target band results were normalized to the expression of cardiac GAPDH.^14^ It was not possible to analyse GAPDH (37 kDa) as a normalizer on the same gel as the ryanodine receptor (565 kDa) due to the difference in molecular weight between the two proteins. Due to this, the ryanodine receptor is expressed without normalization.

## Statistical analysis

Statistical analysis was performed using Sigma Stat 3.5 software (SYSTAT Software Inc., San Jose, CA, USA). The distribution of variables was assessed using the Kolmogorov-Smirnov test for normality. According to the normality of the data, the results are reported as the means ± standard deviation (SD) or median (25th percentile; 75th percentile). Comparisons between groups were performed using two-tailed Student’s t-test for independent samples or Mann-Whitney test or repeated-measures two-way analysis of variance (ANOVA) when appropriate. The level of significance considered was 5%.

Sample size (n) was estimated using the equation for comparison between groups: n = 2SD2(Zα/2 + Zβ)2/d, where n is the sample size, SD = 0.02 from previous studies, Zα/2 = 1.96 (from Z table) at type 1 error of 5%, Zβ = 0.842 (From Z table) at 80% power and d = 0.02 (effect size - minimal difference between mean values).^28^ The sample size needed to detect a significant difference between groups was 16 rats per group; however, we decided to use 22 simulated (sham) and 18 aortic stenosis induction (AoS) animals per group for the study design.

## Results

### Echocardiographic evaluation and heart failure signs

Echocardiogram data revealed that aortic stenosis resulted in predominantly concentric cardiac hypertrophy (↑RWT, ↑PWDT, ↑ISDT, and ↑LVDD), left atrium dilation (↑LA/Ao), and diastolic (↑E wave, ↑E/A, ↑E/E’, ↓E’, and ↓A’) and systolic dysfunction (↑LVSD, ↓PWSV, ↓FS, and ↓EF) 28 weeks after surgery (Table 1). The following clinical and pathological signs of HF were detected: ascites (30%), left atrium thrombi (48%), pleural effusion (68%), tachypnoea (79%), and right ventricular hypertrophy (100%) (Table 1).

**Table 1:** – Echocardiogram data and heart failure signs

	Sham	AoS	p-value
HR (bpm)	302 ± 40	298 ± 40	0.857
LVDD (mm)^*^	7.55 (7.15; 7.66)	8.43 (7.27; 9.20)	<0.001
LVSD (mm)^*^	3.20 (2.81; 3.32)	3.83 (3.32; 5.62)	<0.001
PWDT (mm)^*^	1.53 (1.53; 1.65)	2.81 (2.55; 3.07)	<0.001
ISDT (mm)^*^	1.65 (1.53; 1.70)	3.07 (2.84; 3.26)	<0.001
RWT	0.43 ± 0.03	0.69 ± 0.16	<0.001
LA/Ao	1.22 ± 0.09	1.91 ± 0.18	<0.001
E wave (cm/s)	85 ± 7	132 ± 18	<0.001
E/A	1.49 ± 0.18	5.13 ± 1.40	<0.001
E’ (cm/s)	6.20 ± 0.78	5.32 ± 0.89	<0.001
A’ (cm/s)	4.28 ± 0.67	3.12 ± 1.18	<0.001
E/E’	13.9 ± 2.22	25.2 ± 4.66	<0.001
PWSV (cm/s)	68 ± 9	37 ± 9	<0.001
FS (%)	26.1 ± 3.42	23.3 ± 4.69	0.028
EF (%)^*^	93 (92; 94)	89 (79; 92)	<0.001
Ascites (%)	0	30	-
LAT (%)	0	48	-
PE (%)	0	68	-
Tachypnea (%)	0	79	-
RVH (%)	0	100	-

Data are expressed as means ± SD or median (25 percentile; 75 percentile)^*^. Sham: animals submitted to simulated surgery (n= 28); AoS: animals submitted to aortic stenosis surgery (n= 18). HR: heart rate; LVDD: left ventricle diastolic diameter; LVSD: left ventricle systolic diameter; PWDT: posterior wall diastolic thickness; ISDT: interventricular septum diastolic thickness; RWT: left ventricle relative wall thickness; LA: left atrium; AO: aorta diameter; E/A: ratio between filling flow peak (E wave) and atrial contraction flow peak (A wave); EF: ejection fraction; FS: midwall fraction shortening; PWSV: posterior wall systolic velocity; E’: velocity of the mitral annulus during early ventricular filling; A’: mitral velocity annulus during atrial contraction; E/E’: ratio between filling flow peak and mitral annulus velocity during early ventricular filling. LAT: left atrium thrombi; PE: pleural effusion; RVH: right ventricular hypertrophy. Student’s t-test or Mann-Whitney test. *p < 0.05.

### Isolated papillary muscle evaluation

#### Baseline data

Aortic stenosis impaired myocardial contractile and relaxation function by reducing developed tension and the maximum rate of tension development and increasing rest tension and time-to-peak tension (Table 2).

**Table 2:** – Baseline data

	Sham	AoS	p-value
CSA (mm^2^)	1.15 ± 0.16	1.18 ± 0,20	0.589
DT (g/mm^2^)	6.26 ± 1,58	5.18 ± 0.93	0.039
RT (g/mm^2^)	0.60 ± 0.20	0.80 ± 0.24	0.010
+dT/dt (g/mm^2^/s)	66.6 ± 17.7	46.9 ± 10.3	0.001
-dT/dt (g/mm^2^/s)	22.1 ± 5.24	23.9 ± 5.40	0.346
TPT (ms)^#^	180 (180; 185)	200 (180; 217)	0.007

*Data are expressed as means ± SD or median (25 percentile; 75 percentile)^#^. Sham: animals submitted to simulated surgery (n= 22); AoS: animals submitted to aortic stenosis surgery (n= 12). CSA: papillary cross-sectional area; DT: maximum developed tension; RT: resting tension; +dT/dt: maximum rate of tension development; -dT/dt: maximum rate of tension decline; TPT: time-to-peak tension. Student’s t-test or Mann-Whitney test. p< 0.05.*

#### Isolated papillary muscle manoeuvres

Figure 1A-C presents the papillary muscle response percentage to postrest contraction (10, 30, and 60 s). Animals with aortic stenosis exhibited a poor response to the postrest contraction manoeuvre compared to sham animals for all evaluated time points and variables. There was no significant difference between groups at any time point. Figure 1D-F presents the percent papillary muscle response to increase extracellular calcium concentration (1.5, 2.5, and 3.5 mM). Figure 2A-C shows papillary muscle responses to SERCA2a inhibition and an increase in calcium concentration. After SERCA2a inhibition by cyclopiazonic acid, there was a significant difference between groups in calcium concentration at 0.5 mM for maximum developed tension (Figure 2A). The maximum rate of tension development and decline did not exhibit a difference between the AoS and sham groups. Figure 2D-F presents the papillary muscle response to L-type calcium channel inhibition, demonstrating an increase in calcium concentration. Animals with aortic stenosis had worse performance in response to calcium elevation after L-type calcium channel blockage than sham animals for all evaluated time points and variables.

**Figure 1:** – Response percentage to post-rest contraction (1A, B, and C) and elevation of extracellular calcium concentration (1D, E, and F) from baseline (Ca^2+^ concentration: 0.5 mM). DT: maximum developed tension; +dT/dt: maximum rate of tension development; -dT/dt: maximum rate of tension decline. Data are expressed as means ± SD of maneuver response percentage. Sham: animals submitted to simulated surgery (n= 22); AoS: animals submitted to aortic stenosis surgery (n= 12). Analysis of variance for repeated measures and Bonferroni post-hoc test. *p< 0.05 vs Sham; †p< 0.05 vs. 10 seconds; ‡p< 0.05 vs. 10 seconds and 30 seconds (1A-C); †p< 0.05 vs. 1.5 Ca^2+^; ‡p< 0.05 vs. 1.5 and 2.5 Ca^2+^ (1D-F).

**Figure 2:** – Inhibition percentage of DT (maximum developed tension), +dT/dt (maximum rate of tension development), and –dT/dt (maximum rate of tension decline) to cyclopiazonic acid (SERCA2a blocker; figure 2A, B, and C) and diltiazem (L-type calcium channels blocker; figure 2D, E, and F) plus incremental calcium concentration. Data are expressed as means ± SD of maneuver response percentage. Sham: animals submitted to simulated surgery (n= 22); AoS: animals submitted to aortic stenosis surgery (n= 12). Analysis of variance for repeated measures and Bonferroni post-hoc test. *p< 0.05 vs Sham; †p< 0.05 vs. 0.5 Ca^2+^; ‡p< 0.05 vs. 0.5, and 1.5 Ca^2+^; §p< 0.05 vs. 0.5, 1.5, and 2.5 Ca^2+^.

## Isolated cardiomyocytes

### Mechanical function and calcium handling analysis

Figure 3A-F shows cardiomyocyte mechanical function. Aortic stenosis impaired shortening maximum velocity (Figure 3B) and times to achieve 50% cardiomyocyte contraction (Figure 3E) and relaxation peak (Figure 3F). Figure 3G-J summarizes calcium handling 28 weeks after aortic stenosis. AoS animals presented alterations in both the time to achieve Ca^2^ peak and time to 50% Ca^2^ decay.

**Figure 3:** – Cardiomyocytes mechanical function and cardiomyocyte calcium handling. SMV: shortening maximum velocity; RMV: relaxation maximum velocity; TS_50%_: time to 50% shortening; TR_50%_: time to 50% relaxation; TCD_50%_: time to 50% of Ca^2+^ decay. Data are expressed as means ± SD or median (25 percentile; 75 percentile). Sham: animals submitted to simulated surgery (n= 6; number of cells= 36); AoS: animals submitted to aortic stenosis surgery (n= 6; number of cells= 36). Student’s t-test. *p< 0.05.

### Expression of calcium handling proteins

Data regarding protein expression of calcium handling regulator elements are shown in Figure 4A-E. Aortic stenosis increased L-type calcium channel, SERCA2a, and Na^+^/Ca^2^exchanger (NCX) protein expression and decreased phosphorylation of the Thr(17) residue of PLB.

**Figure 4:** – Expression of calcium handling protein. Data are expressed as means ± SD. Sham: animals submitted to simulated surgery (n= 7); AoS: animals submitted to aortic stenosis surgery (n= 7). LTCC’s: L-type calcium channels; SERCA2a: Sarco/endoplasmic reticulum Ca^2+^; PLB: Phospholamban; PLB_ser16_: phosphorylated phospholamban at serine 16; PLB_thr17_: phosphorylated phospholamban at threonine 17; RyR: Ryanodine; NCX: Na+/Ca2+ exchanger. Ryanodine is expressed without normalization. Student’s t-test. *p< 0.05.

## Discussion

In the experimental model of supravalvular aortic stenosis, studies investigating pathologic remodelling and HF, focusing on specific Ca^2^ handling alterations and their regulatory elements, have presented scattered data and a poor mechanistic reflection. As such, this work performed an overall evaluation of cardiac function, Ca^2^ cellular dynamics, and Ca^2^ regulatory elements to elucidate the dysfunctional process of the major components responsible for Ca^2^ balance and its influence on cardiac function in HF induced by aortic stenosis.

In this study, aortic stenosis promoted structural changes and ventricular dysfunction, both diastolic and systolic, as assessed by echocardiogram, similar to previous studies.^14,15,17,29^ AoS animals developed concentric left ventricular hypertrophy and left atrial dilation, striking characteristics in this experimental cardiac pressure overload model.^1,14,15,17,29^ Based on Laplace’s law (Stress = Pressure x Radius/2 x Thickness), the increase in the left ventricular relative wall thickness was intended to normalize systolic parietal stress due to mechanical aortic obstruction.^1,34,35^ However, the decrease in systolic function suggests that even after the hypertrophic process, the systolic parietal stress had likely normalized, and the decrease in contractile capacity was responsible for the depression of systolic performance. The mechanical function of the papillary muscles and cardiomyocytes reproduced similar responses to the echocardiographic examination. AoS animals presented a decreased and slower ability to develop force (↓DT and +dT/dt), to shorten (↓SMV and ↑TS_50%_) and to relax (↑RT and TR_50%_). This functional damage to the isolated papillary muscle in the late stage of aortic stenosis is in line with our previous study, which analysed heart disease six weeks after surgery.^16^ In addition, in agreement with the isolated cardiomyocyte results, data from the literature show that there is a decrease in the shortening velocity of cardiomyocytes.^18^ In our animals, functional cardiac depression resulted in severe HF, expressed by the following clinical and pathological signs: altered breathing pattern, ascites, pleural effusion, and atrial thrombus.

In this experimental model, the pathological process of *maladaptive* remodelling may induce an oxygen deficit as a starting point. Myocardial capillary rarefaction,^22^ a marked ventricular wall hypertrophy product, may represent the origin of pathology. Several mechanisms may be enacted and hyperactivated to readjust the cardiac structural and functional pattern, including sympathetic tonus, the renin-angiotensin-aldosterone system, inflammatory mediators, oxidative stress, and the regulation of myocardial gene expression via microRNAs.^36^ However, the disharmonic activation of these microsystems due to cardiac and body demands generates numerous pathophysiological responses, including impairments in the calcium handling of cardiomyocytes.^1,34^

Mismatch of cytosolic Ca^2^ in cardiomyocytes is one of the key mechanisms for cardiac malfunction in response to various injury types.^1,4,39^ In HF models, including experimental aortic stenosis, researchers have characterized changes in transmembrane expression and function, as well as the intracellular proteins that regulate Ca^2^ handling.^18,20,21,43^ In the present study, the increase in SERCA2a and NCX protein expression may have been an adaptive response to decrease or avoid cytosolic Ca^2^ overload at the end of diastole. This response was partially efficient since there was impairment of time for cytosolic calcium decay in the isolated cardiomyocytes; in addition, even with the molecular compensatory pattern, diastolic functional impairment was verified a reduced 50% relaxation time (TR_50%_). As in this study, the literature shows an increase in NCX protein expression in HF.^18,44^ However, our results differ with respect to SERCA2a,^16,17^ which, generally, is unchanged or decreased in this pathological scenario.^18,44,45^ It is important to note that slow Ca^2^ reuptake may decrease the concentration of this ion in the sarcoplasmic reticulum (SR) over time.

Consequently, there is a decrease in the amount available for release,^4,44^ via RyR, during systole in the Ca^2^ release mechanism induced by Ca^2^ from LTCC. In the present study, we observed increased LTCC protein expression in animals with heart disease. However, this adaptive process seems to have been inefficient since the animals’ cardiomyocytes slowed the time to achieve peak Ca^2^ with consequent reduction of the shortening maximum velocity and increase in the time to reach 50% of the shortening. Converging with the discussion above, Szymanska et al.^12^ proposed that in HF due to aortic stenosis, there are changes in both the uptake and release of Ca^2^ by the SR and that these factors may contribute to deterioration of the processes of cardiac contraction and relaxation.

The manoeuvres in the isolated papillary muscle assay were performed to ascertain the physiological damage of two of the main elements of Ca^2^ dynamics in this pathological process, SERCA2a and LTCC. Cyclopiazonic acid (CPA) blockade and postrest contraction was used to evaluate the potential for Ca^2^ reuptake and the functional capacity of SERCA2a. There was a difference in response between the two manoeuvres to analyse the function of SERCA2a. Post-rest contraction showed that the potential for Ca^2^ reuptake was impaired by aortic stenosis. However, after SERCA2a blockade by CPA, the sham and AoS groups showed similar responses to the variables studied in the analysis. Considering that the cardiopathy animals exhibited higher expression of the referenced protein, the blocking percentage must have been higher in the sham group due to having a lower number of SERCA2a than the AoS group. However, as the number of unblocked proteins in the AoS group was higher than in the sham group, it is possible to infer that this remaining group of SERCA2a postblock AoS animals demonstrated functional impairment.

Our data agree with previous studies that suggest aortic stenosis as an inducer of SERCA2a functional deterioration.^11,12,16^ Furthermore, our findings show that increased expression of SERCA2a, the primary maintainer of cytosolic Ca^2^ homeostasis, was not sufficient to compensate for the decrease in intrinsic activity of this protein; this hypothesis is reinforced by the results of this study, which demonstrated a diminished time to achieve 50% calcium decay in cardiomyocytes. Because SERCA2a is an ATPase, under conditions of low ATP, the intrinsic activity of this protein could be impaired, causing disrupted Ca^2^ reuptake by the SR.^45^ In favour of this hypothesis, a previous study by our group (these results are not published) showed that AoS animals, after two weeks of surgery, present an increase in hypoxia-inducible factor- alpha (HIF-1α), the most important indicator of tissue oxygen deficit, which may indicate a reduction in the production of adenosine triphosphate (ATP).

In addition to the alterations mentioned above, this study identified a decrease in phosphorylation of phospholambam at threonine 17, suggesting that the impairment of Ca^2^ reuptake may not be attributed only to the intrinsic functional impairment of SERCA2a but also to the increased blockage of this protein by phospholambam.

Diltiazem blockade and elevation of extracellular Ca^2^ in the papillary muscle showed that animals with heart disease had impaired LTCC function. Although there was an increase in the protein expression of these channels, there was an increase in the time to achieve the Ca^2^peak in the isolated cardiomyocytes, a reduction in SMV and an increase in TS_50%_. These findings agree with the body of evidence in the literature, which shows that cardiac injury by AoS generates a foetal splicing variant (Ca_v_1.2_e21+22_) that reduces the expression and activity of these channels and increases the ubiquitination of LTCCs via proteasomal degradation.^21^ Furthermore, in pathological remodelling by AoS coupled with decreased I_Ca_, there is inefficiency in the coupling of LTCCs with RyR receptors, both due to the degradation of T tubules and the decrease in junctophillin-2, the protein responsible for anchoring the sarcoplasmic reticulum to the cell membrane.^20^

### Limitations of the study

In this study, cardiomyocytes were not isolated only from the left ventricle. Thus, the results of cardiomyocytes from both the left and right ventricles were analysed and discussed. It is important to highlight that a proper understanding of cardiac physiology in cellular studies requires ventricular contractility knowledge since the right and left ventricles have distinct functional properties. In this regard, it would also be relevant to perform molecular analysis in both ventricles; however, the expression of calcium handling proteins was only performed in the LV.

## Conclusions

Our study sought to clarify and facilitate understanding of the pathophysiological events in Ca^2^ handling and changes in its primary regulatory agents in the cardiac pathological process due to aortic stenosis. According to our results, in this HF experimental model, there are essential changes in calcium dynamics due to alterations in NCX and SERCA2a expression, L-type calcium channel protein expression, and reduced phosphorylation of the Thr(17) residue of PLB. Furthermore, SERCA2a and L-type calcium channel functional impairment were fundamental for contractile and relaxation deterioration. Therefore, it is important to develop therapies that focus not only on SERCA2a and L-type calcium channels but also on understanding all pathological processes to rebalance intracellular calcium flow and cardiac function.
